# The *Chlamydia pneumoniae* inclusion membrane protein Cpn0308 interacts with host protein ACBD3

**DOI:** 10.1128/jb.00275-24

**Published:** 2024-12-26

**Authors:** Liang Ma, Xiao-hui Jia, Zhe Gao, Yan Zhou, Yong-ting Cheng, Ping Li, Tian-jun Jia

**Affiliations:** 1 Pathogen Biology and Immunology Research Institute, Hebei North University 261761 , Zhangjiakou, Hebei, China; 2 Key Laboratory of Clinical Laboratory Diagnostics, Hebei North University 261761 , Zhangjiakou, Hebei, China; 3 Handan Vocational College of Science and Technology, Han Dan, Hebei, China; Queen Mary University of London, London, United Kingdom

**Keywords:** Cpn0308, ACBD3, inclusion membrane proteins, co-immunoprecipitation, GST pull-down, subcellular localization

## Abstract

*Chlamydia pneumoniae* is an obligate intracellular bacterium of eukaryotic cells characterized by a unique biphasic life cycle; its biosynthesis and replication must occur within a cytoplasmic vacuole or inclusion. Certain inclusion membrane proteins have been demonstrated to mediate the interactions between intra-inclusion chlamydial organisms and the host cell. It has been demonstrated previously that the *C. pneumoniae*-encoded Cpn0308 localizes to the inclusion membrane; however, its function remains unknown. In the current study, a yeast two-hybrid assay was conducted to screen Cpn0308 as a bait against a HeLa cell cDNA library, revealing its binding to the host protein acyl-coenzyme A binding domain-containing 3 (ACBD3). The interaction between Cpn0308 and ACBD3 was confirmed through co-immunoprecipitation and GST (Glutathione S-transferase) pull-down assays. The two proteins were also co-localized in HeLa cells co-expressing Cpn0308 and ACBD3, as well as in *C. pneumoniae*-infected cells, as observed under confocal fluorescence microscopy. Given that ACBD3 plays a crucial role in maintaining host cell lipid homeostasis and its Golgi dynamic domain is responsible for interacting with Cpn0308, we hypothesize that the Cpn0308-ACBD3 interaction may facilitate *C. pneumoniae*’s acquisition of host lipids, thereby benefiting chlamydial survival. This study lays a foundation for further elucidating the mechanisms of Cpn0308-mediated *C. pneumoniae* pathogenesis.

**IMPORTANCE:**

The biosynthesis and replication of *Chlamydia pneumoniae* (*Cpn*) must occur within the cytoplasmic vacuoles or inclusions of host cells. Inclusion bodies play a crucial role in mediating the interactions between *Cpn* and host cells. Cpn0308 is localized to the inclusion membrane; however, its function is unknown. In this study, Cpn0308 was found to bind to host protein acyl-coenzyme A binding domain-containing 3 (ACBD3) through some standard approaches. Co-localization of the two proteins was observed in both original HeLa cells and Cpn-infected HeLa cells. ACBD3 plays a significant role in maintaining lipid homeostasis in host cells; we speculate that the Cpn0308-ACBD3 interaction may facilitate the acquisition of host lipids by *C. pneumoniae*, thereby enhancing chlamydial survival.

## INTRODUCTION

*Chlamydia pneumoniae* is a ubiquitous pathogen associated with various respiratory diseases, including pneumonia, bronchitis, asthma, and pharyngitis (1–6). Similar to other chlamydial species, *C. pneumoniae* exhibits a typical biphasic developmental cycle (7), alternating between an infectious elementary body (EB) and a noninfectious reticulate body (RB). After entering a host cell, an EB undergoes differentiation into an RB within a membrane-bound cytoplasmic vesicle known as an inclusion (8). Inclusions safeguard the differentiation of EB into RB, the biosynthesis and replication of RB, and the conversion of progeny RB back to EB (9–11). Intra-inclusion chlamydial organisms produce numerous proteins that are inserted into the inclusion membrane, referred to as inclusion membrane proteins (Incs), to mediate chlamydial interactions with host cells (12). Incs have been demonstrated to shield the chlamydial inclusions from host immune attack and facilitate the acquisition of nutrients by chlamydia from host cells (13, 14). Although the hypothetical protein Cpn0308 was previously identified as an Inc (15, 16), its host cell interaction partners remain unidentified, and its functions have yet to be determined.

In the present study, we identified the host cell protein acyl-coenzyme A binding domain-containing 3 (ACBD3) as a binding partner of Cpn0308 using the yeast two-hybrid (Y2H) method. The interaction of Cpn0308 with ACBD3 was validated through co-immunoprecipitation, GST pull-down assays, and fluorescence microscopy. Cpn0308 binds to the Golgi dynamic (GOLD) region of ACBD3. These findings lay a foundation for further investigation into the functional consequences of the Cpn0308-ACBD3 interaction.

## MATERIALS AND METHODS

### Y2H screening of Cpn0308 against a HeLa cDNA library

A Y2H screening was conducted using a Matchmaker GAL4 two-hybrid system (cat#630489, Clontech, Beijing, China). To construct the bait, the Cpn0308 gene was amplified from the genomic DNA of *C. pneumoniae* AR39 (provided by Dr. Guangming Zhong, University of Texas Health Science Center at San Antonio) using PCR (all primer sequences in this study are listed in Table 1). The PCR fragment was subsequently inserted into the plasmid pGBKT7 to generate recombinant bait plasmid pGBKT7-Cpn0308. After confirming that the pGBKT7-Cpn0308 bait plasmid exhibited no self-activation and toxicity on yeast strains AH109 and Y187, Y2H was performed between the bait yeast strain AH109 (transformed with pGBKT7-Cpn0308) and the library yeast strain Y187 transformed with pGADT7 containing the HeLa cell cDNA library (cat#342189, Be Na Culture Collection, He Nan, China). The colonies were screened using β-galactosidase staining and colony PCR assays and were further confirmed through backcross experiments. The screened plasmids were sequenced, and the prey sequences were used to BLAST search for homologous proteins in National Center for Biotechnology Information (NCBI.

**TABLE 1:** The primer sequences of the study

Primer name	Sequence(5′−3′)
pGBKT7-Cpn0308 forward primer	TCCGAATTCATG GCTAC AGTAGCACAAAC (*EcoR* I)
pGBKT7-Cpn0308 reverse primer	GCGGGATCCTTATTTAGAGGAGTAACGAT (*Bam*H I)
ACBD3 gene forward primer	GGGGTACCAGGAAGGAGGA AGAGGAGCG(*Kpn* I)
ACBD3 gene reverse primer	CCGGAATT CCAGCAAAGGCTTGTTGGCA(*EcoR* I)
pcDNA3.1/Myc-His-Cpn0308 pGEX-6P-2-Cpn0308 forward primer	CGCGGATCCATGGCTACAGTAGCACAAACA(*Bam*H I)
pcDNA3.1/Myc-His-Cpn0308 pGEX-6P-2-Cpn0308 reverse primer	TTTTCCTTTTGCGGCCGCTTATTTAGAGGAGTAACGAT(*Not* I)
ACBD3 (100–184) forward primer	CGC-GGATCC-GCCACCATGAAAGCATTTCATCCAACTTATGAAG(*Bam*H I)
ACBD3 (100–184) reverse primer	TTTTCCTTTT-GCGGCCGC-TTCCTTCTCTATTTTGTGGGACG(*Not* I)
ACBD3 (100–245) forward primer	CGC-GGATCC-GCCACCATGAAAGCATTTCATCCAACTTATGAAG(*Bam*H I)
ACBD3 (100–245) reverse primer	TTTTCCTTTT-GCGGCCGC-CTGCTGCTTTTGCTGCTCC(*Not* I)
ACBD3 (246–528) forward primer	CGC-GGATCC-GCCACCATGATAATGGCAGCTTTAAACTC(*Bam*H I)
ACBD3 (246–528) reverse primer	TTTTCCTTTT-GCGGCCGC-TCTAGTATAATAGACTCTGTAG(*Not* I)
ACBD3 (400–528) forward primer	CGC-GGATCC-GCCACCATGGGAGAAGTGGTCACTGTT(*Bam*H I)
ACBD3 (400–528) reverse primer	TTTTCCTTTT-GCGGCCGC-TCTAGTATAATAGACTCTGTAG(*Not* I)

### Recombinant expression vector construction

Three expression vectors were constructed to express recombinant proteins. The ACBD3 gene was amplified using PCR. The digested PCR products were ligated to pcDNA3.1+/Flag plasmid, which had been digested with the same set of restriction enzymes, to construct the recombinant eukaryotic expression plasmid pcDNA3.1+/Flag-ACBD3. The recombinant plasmids pcDNA3.1/Myc-His-Cpn0308 and pGEX-6P-2-Cpn0308 were constructed in a similar manner. The eukaryotic expression plasmids pcDNA3.1+/Flag-ACBD3 and pcDNA3.1/Myc-His-Cpn0308 were transfected into HeLa cells using the Lipofectamine 2000 transfection reagent (17), while the prokaryotic expression plasmid pGEX-6P-2-Cpn0308 was transformed into *Escherichia coli* XL1-Blue competent cells for protein (18).

### Co-immunoprecipitation assay

The recombinant plasmids pcDNA3.1/Myc-His-Cpn0308 and pcDNA3.1+/Flag-ACBD3 were co-transfected into HeLa cells. After a 48-hour culture, the cells were collected and lysed. The lysate was incubated with Pierce Anti-c-Myc agarose beads (cat#20168, Thermo Fisher, Beijing, China) overnight at 4°C. After washing with phosphate-buffered salin solution, the bead antibody-immobilized antigens were resolved by SDS-PAGE, and the antigen bands were transferred onto a nitrocellulose membrane for detection with antibodies following a standard western blot protocol as described previously (19). Briefly, the nitrocellulose membranes were incubated in a blocking buffer for 2 hours at room temperature. Then, mouse anti-Flag antibody (cat#F1804, Sigma, Shanghai/China) and/or rabbit anti-c-Myc antibody (cat#bs-24507R, Bioss, Beijing, China) were used as primary antibodies. The primary antibody binding was probed with goat anti-rabbit IgG and/or goat anti-mouse IgG antibodies conjugated with horseradish peroxidase and visualized using ECL (cat#bs-0295G/bs-0296G, Bioss, Beijing, China).

### GST pull-down assay

The Pierce GST Protein Interaction Pull-Down Kit (cat#21516, Thermo Fisher, Beijing, China) was utilized to perform the GST pull-down assay. GST-Cpn0308 served as the bait protein, while ACBD3 functioned as the prey protein. The glutathione bead-immobilized bait protein GST-Cpn0308 was employed to capture the prey protein. After washing away unbound proteins, the pellets were resolved by SDS-PAGE, and the gel-resolved protein bands were detected using western blotting as described above. To identify the binding site of Cpn0308 in ACBD3, the prey protein ACBD3 was expressed in four different fragments, including ACBD3 (100–184), ACBD3 (100–245), ACBD3 (246–528), and ACBD3 (400–528), using the expression vector pEnCMV-MCS-3×Flag as described in the manual (20). The primer sequences used for cloning each of the four ACBD3 fragments into the expression vector are presented in Table 1.

### Immunofluorescence microscopy

The recombinant plasmids pcDNA3.1/Myc-His-Cpn0308 and pcDNA3.1+/Flag-ACBD3 were co-transfected into HeLa cells. After a 24-hour culture, the cells were fixed with acetone. Following blocking, rabbit anti-Myc (cat#bs-24507R, Bioss, Beijing, China) and mouse anti-Flag (cat#F1804, Sigma, Shanghai, China) antibodies were utilized as primary antibodies to detect the corresponding tags expressed in HeLa cells. The primary antibody binding was probed with goat anti-mouse IgG-Cy3 (cat#bs-0296G-Cy3, Bioss, Beijing, China) and/or goat anti-rabbit IgG-FITC antibodies (cat#bs-0295G-FITC, Bioss, Beijing, China) and visualized under a laser scanning confocal microscope (cat#A1SiR, Nikon, Tokyo, Japan). To improve image quality and provide a more comprehensive perspective, images were observed and captured in layers with a gradient of 1 µm. To localize endogenous ACBD3, an anti-ACBD3 antibody was used as the primary antibody (cat#14096–1-AP, Proteintech, Chicago, Illinois, USA). To localize Cpn0308 secreted by *C. pneumoniae*, *C. pneumoniae*-infected HeLa cells were labeled using an anti-Cpn0308 primary antibody (provided by Dr. Guangming Zhong, University of Texas Health Science Center at San Antonio).

### Bioinformatics Analysis

The three-dimensional structures of Cpn0308 and ACBD3 were predicted using the *de novo* prediction method provided by the Rosetta online server (http://robetta.bakerlab.org). Molecular dynamic optimization of the constructed models was performed using GROMACS software. To assess the reliability of the two models, Ramachandran plot data analysis was conducted using the PROCHECK program from the SAVES v6.0 server of UCLA-DOE LAB(https://saves. mbi.ucla.edu/). The optimized structural models of Cpn0308 and ACBD3 were docked using Vakser Lab online software (https://vakserlab.ku.edu/) to create the docking models of the Cpn0308-ACBD3 complex. The bonding distances of hydrogen bonds and salt bridges in the Cpn0308-ACBD3 docking complex were analyzed using the PDBePISA online server (https://www.ebi.ac.uk/pdbe/pisa/), and the amino acid residues involved in the interaction between the two molecules were predicted. The final results were analyzed using PyMol 2.5.1 software.

### Enzyme-linked immunosorbent assay

The Cpn0308-ACBD3 interactions were also measured using enzyme-linked immunosorbent assay (ELISA). The 96-well plate was coated with each of the four ACBD3 protein fragments, prepared as described above, by adding 100 µL of each protein sample (4.0 µg/mL) to each well and incubating overnight at 4°C. After blocking, the GST-Cpn0308 fusion protein was co-incubated with the ACBD3 fragments. After washing, the immobilized GST-Cpn0308 was detected using a mouse anti-GST antibody (cat#66001–1-Ig, proteintech, Chicago, Illinois, USA) and visualized with a goat anti-mouse antibody conjugated with HRP (cat#bs-0296G-HRP, Bioss, Beijing, China), along with a soluble substrate (cat#C04-03002, Bioss, Beijing, China). The color change of the substrate was measured at 405 nm and recorded as optical density (OD) values using an ELISA plate reader (cat#MK3, Thermo Fisher, Waltham, Massachusetts, USA). To ensure that the immobilized GST is due to the binding of Cpn0308 to ACBD3 or its fragments, a free GST protein was similarly co-incubated with the ACBD3 fragments as negative controls. In some wells, the GST-Cpn0308 protein was directly coated onto the microplate as a positive control.

## RESULTS

### Y2H screening of Cpn0308 against a HeLa cDNA library identifies the host protein ACBD3 as a binding partner

**Fig 1:**
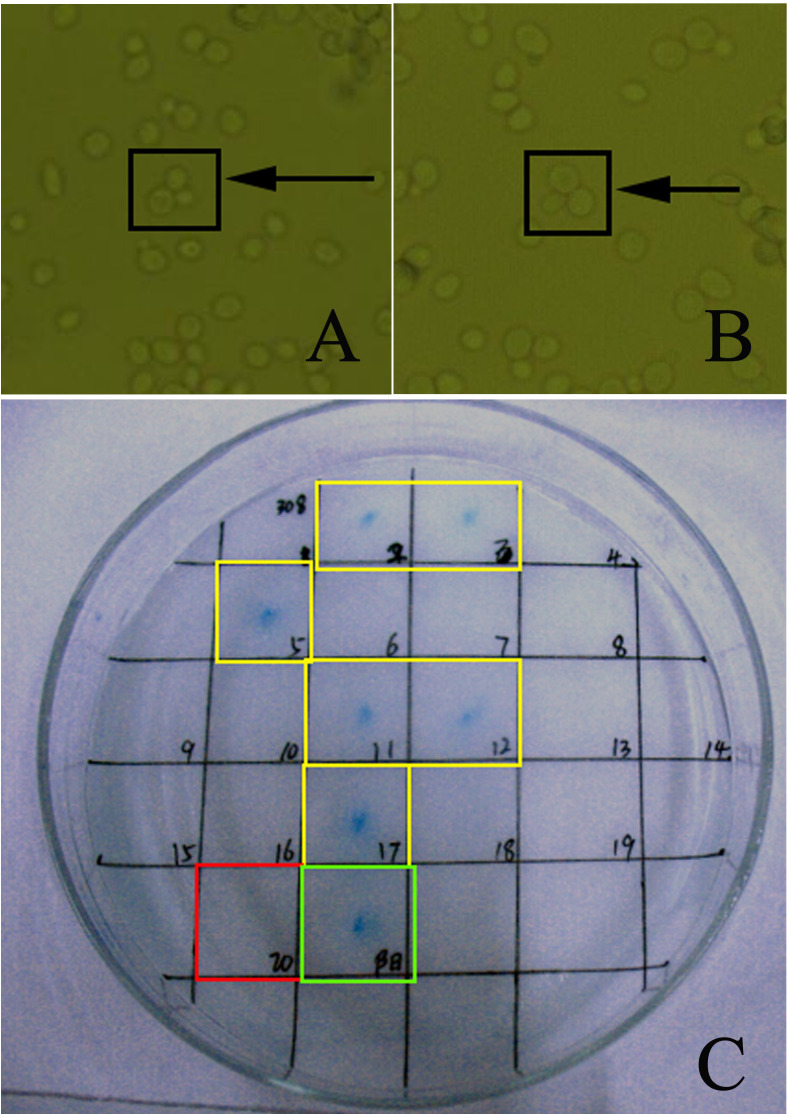
Y2H to screen the ligands interacting with Cpn0308. The pGBKT7-Cpn0308-transformed yeast strain AH109 was co-cultured with the strain Y187, containing a GAL4AD fusion cDNA library derived from HeLa cells for 16 hours at 30°C.The yeast zygotes were observed as distinctive clover-like structures. Representative microscopic images of yeast zygotes from experimental group (**A**) and positive control group (**B**) were shown. A plate image showed β-galactosidase staining-positive colonies including #2, 3, 5, 11, 12, and 17 (yellow rectangular frame), while #20 is a negative control (red rectangular frame) and #21 a positive control colony (green rectangular frame) (**C**). PCR was used to detect plasmids from positive clones #2, 3, 5, 11, 12, and 17.

A mixed culture of yeast strain AH109 transformed with pGBKT7-Cpn0308 and strain Y187 containing a GAL4 AD fusion cDNA library from HeLa cells was conducted, and the typical clover-like structure (yeast zygote) was observed. The interaction between the bait protein and the protein expressed from the library activated the downstream reporter gene LacZ, leading to the synthesis of galactosidase, which produced visible blue colonies. A total of six positive colonies were generated, and plasmids were extracted from the blue colonies; subsequently, the genes coding for potential binding partners were sequenced. NCBI searches consistently led to the identification of ACBD3 as the potential binding partner of the bait Cpn0308 (Fig. 1).

### Cpn0308 interacts with ACBD3 in both co-immunoprecipitation and GST pull-down assays

**Fig 2:**
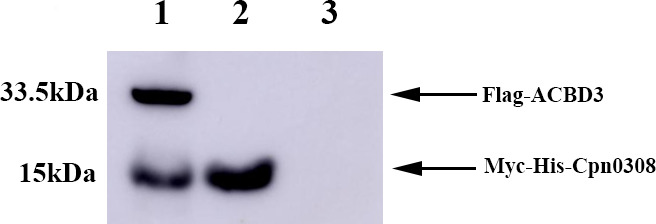
Immunoprecipitation of the Cpn0308-ACBD3 interaction complexes. The lysates of HeLa cells co-transfected with both pcDNA3.1/Myc-His-Cpn0308 and pcDNA3.1+/Flag-ACBD3 (lane 1), the pcDNA3.1/Myc-His-Cpn0308 plasmid alone (lane 2), or both the pcDNA3.1/Myc-His vector and pcDNA3.1+/Flag-ACBD3 plasmids (lane 3) were precipitated using Myc antibody beads. The precipitates were resolved in SDS-PAGE, and the resolved protein bands were detected using antibodies against Myc and Flag tags, respectively. The Myc-His-Cpn0208 fusion protein migrated at 15 kDa while the Flag-ACBD3 fusion protein at 33.5 kDa. The complete original experimental image can be found in Fig. S1 (Supplementary File).

To confirm the interaction between the chlamydial protein Cpn0308 and the host cell protein ACBD3, a co-precipitation assay was performed as described previously (21). Myc-specific antibody-conjugated beads were used to precipitate the lysates made from HeLa cells co-transfected with pcDNA3.1/Myc-His-Cpn0308 and pcDNA3.1+/Flag-ACBD3. The SDS-PAGE-resolved precipitates were probed with anti-Flag and anti-Myc antibodies in a western blot analysis (Fig. 2). The bait Myc-Cpn0308 protein was detected as a band migrating at 15 kDa, while the co-precipitated Flag-ACBD3 protein was detected as a 33.5-kDa band. Both Myc-Cpn0308 and Flag-ACBD3 were detected in the precipitate obtained from the co-transfected HeLa cells (lane 1). As quality controls for the precipitation assay, only the 15-kDa bait protein was detected in the cells transfected with the pcDNA3.1/Myc-His-Cpn0308 plasmid alone (lane 2), while no band was detected in cells expressing Flag-ACBD3 and Myc without Cpn0308 (lane 3). These controls validated that the co-precipitation of ACBD3 was dependent on the interaction of Cpn0308 with ACBD3.

**Fig 3:**
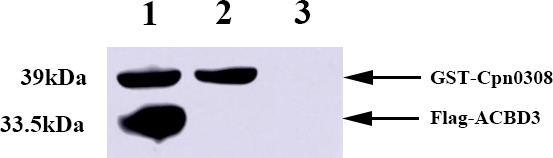
GST-Cpn0308 pull-down of ACBD3. The GST-Cpn0308 fusion protein purified from *E. coli* harboring the pGEX-6P-2-Cpn0308 plasmid was used as a bait to incubate with the prey protein Flag-ACBD3. The pull-down pellets were resolved in SDS-PAGE and detected with antibodies against Myc and Flag in a western blot. Lane 1 was loaded with the pellet pulled down from the GST-Cpn0308 and Flag-ACBD3 co-incubation. Lane 2 from the GST-Cpn0308 bait alone while lane 3 with ACBD3 alone. The GST-Cpn0308 band migrated at 39 kDa while Flag-ACBD3 at 33.5 kDa. The complete original experimental image can be found in Fig. S2 (Supplementary File).

To independently validate the Cpn0308-ACBD3 interaction, we also performed a GST pull-down assay (Fig. 3). The GST-Cpn0308 fusion protein purified from the pGEX-6P-2-Cpn0308 plasmid-transformed *E. coli* was used as the bait to pull down the Flag-ACBD3 fusion protein. The SDS-PAGE-resolved pellets were probed with anti-Myc and anti-Flag antibodies in a western blot. The GST-Cpn0308 bait protein was detected as a 39-kDa band, while Flag-ACBD3 was detected as a 35-kDa band. Both the bait and prey protein bands were detected in the pellet pulled down from the mixture of GST-Cpn0308 and Flag-ACBD3 (lane 1), while only Cpn0308 (lane 2) was detected in the sample containing the GST-Cpn0308 alone. More importantly, no protein band was detected in the sample containing only the prey Flag-ACBD3 (lane 3). Together, these results demonstrate that the GST pull-down of Flag-ACBD3 is dependent on the interaction between Cpn0308 and ACBD3.

### Cpn0308 co-localizes with ACBD3 in HeLa cells under immunofluorescence microscopy

**Fig 4:**
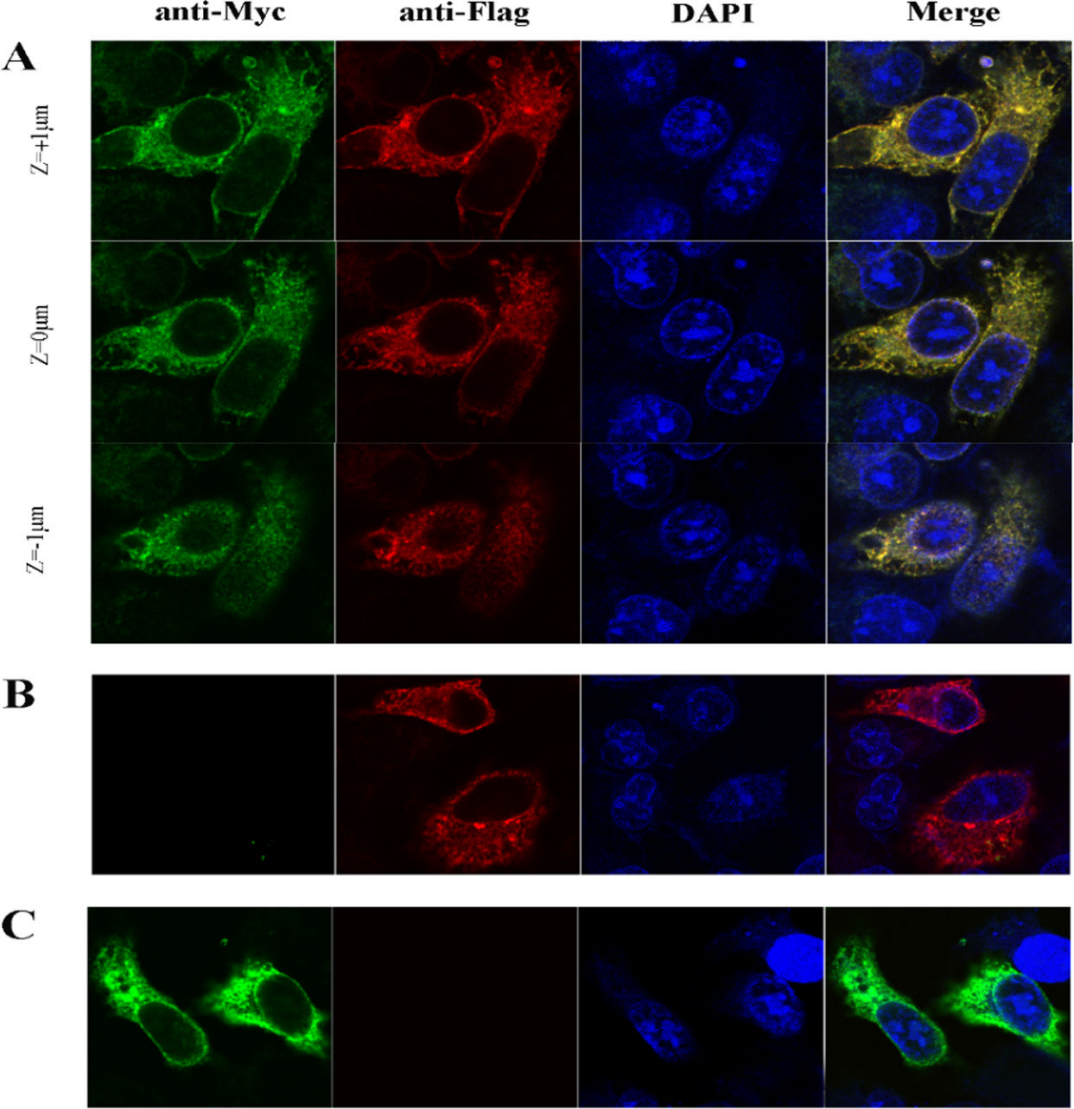
Immunofluorescence localization of Cpn0308 and ACBD3 in transfected HeLa cells. HeLa cells were transfected with both Myc-Cpn0308 and Flag-ACBD3 plasmids (**A**), Myc vector and Flag-ACBD3 (**B**), or Myc-Cpn0308 and Flag vector (**C**). The transfected cells were labeled with a mouse anti-Myc antibody to localize Cpn0308 (green) and a rabbit anti-Flag antibody to detect ACBD3 (red).

The interaction of Cpn0308 with ACBD3 was visualized in HeLa cells co-transfected with pcDNA3.1/Myc-His-Cpn0308 and pcDNA3.1+/Flag-ACBD3 using both anti-Myc (green) and anti-Flag (red) antibodies. As shown in Fig. 4, the overlap (yellow) of green and red fluorescence was detected in HeLa cells co-transfected with both plasmids but not with either plasmid alone. These results indicate that the Myc-Cpn0308 protein co-localizes with Flag-ACBD3. The distribution pattern of the plasmid-encoded proteins is similar to that of the endoplasmic reticulum (ER), which is consistent with the ER distribution pattern of other chlamydial Incs encoded by transgenes (19). The successful co-localization of the transgene-encoded Cpn0308 and ACBD3 has prompted us to further investigate whether *C. pneumoniae*-secreted Cpn0308 co-localizes with the endogenous ACBD3.

### The *C. pneumoniae*-secreted Cpn0308 is co-localized with endogenous ACBD3

**Fig 5:**
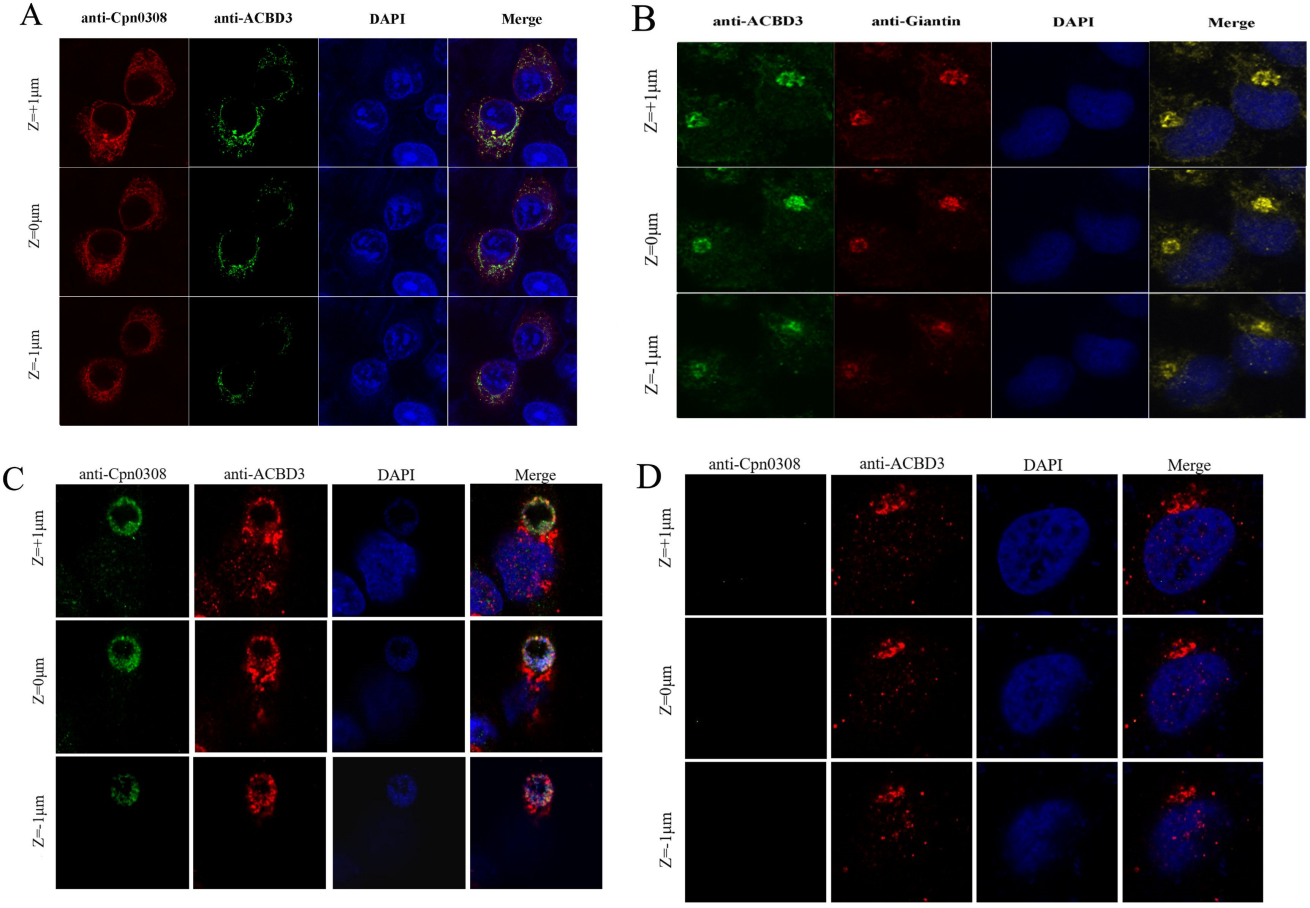
Immunofluorescence co-localization of endogenous ACBD3 with plasmid-expressed or *C. pneumoniae*-secreted Cpn0308. (**A**) HeLa cells were transfected with (**A**) or without (**B**) recombinant plasmid Myc-Cpn0308 or infected with (**C**) or without (**D**) *C. pneumonaie*. The cell samples were immunolabeled with antibodies against Cpn0308 (red) and ACBD3 (green, panel A), or Golgi protein giantin (red) plus ACBD3 (green, panel B). The Cpn0308 was visualized in green and ACBD3 in red (C and D). Images were obtained at 1-µm intervals along the *z*-axis. Please note that the endogenous ACBD3 was localized in the Golgi (**B**) and co-localized with the Cpn0308 either expressed by the recombinant plasmid (**A**) or secreted by *C. pneumoniae* (**C**).

Having demonstrated the interaction between Cpn0308 and ACBD3 in both cell-free assays and inside HeLa cells, we next tested whether Cpn0308 could interact with the endogenous ACBD3 in *C. pneumoniae*-infected cells (Fig. 5). Endogenous ACBD3 was first co-localized with Myc-Cpn0308 in HeLa cells transfected with the recombinant plasmid pcDNA3.1/Myc-His-Cpn0308 (A). Giantin, a Golgi-associated protein, was used to label the Golgi apparatus. Panel B demonstrates the binding of Cpn0308 to the endogenous Golgi (marked by giantin) in HeLa cells transfected with Cpn0308. Panel C shows the co-localization of Cpn0308 with endogenous ACBD3 in HeLa cells infected with *C. pneumoniae*, where ACBD3 is observed to be recruited near *Chlamydia* inclusion bodies. Panel D serves as a negative control without *C. pneumoniae* infection. Thus, we have demonstrated that *C. pneumoniae*-secreted Inc Cpn0308 can interact with host cell ACBD3 during infection.

### The GOLD domain of ACBD3 is responsible for interacting with Cpn0308

**Fig 6:**
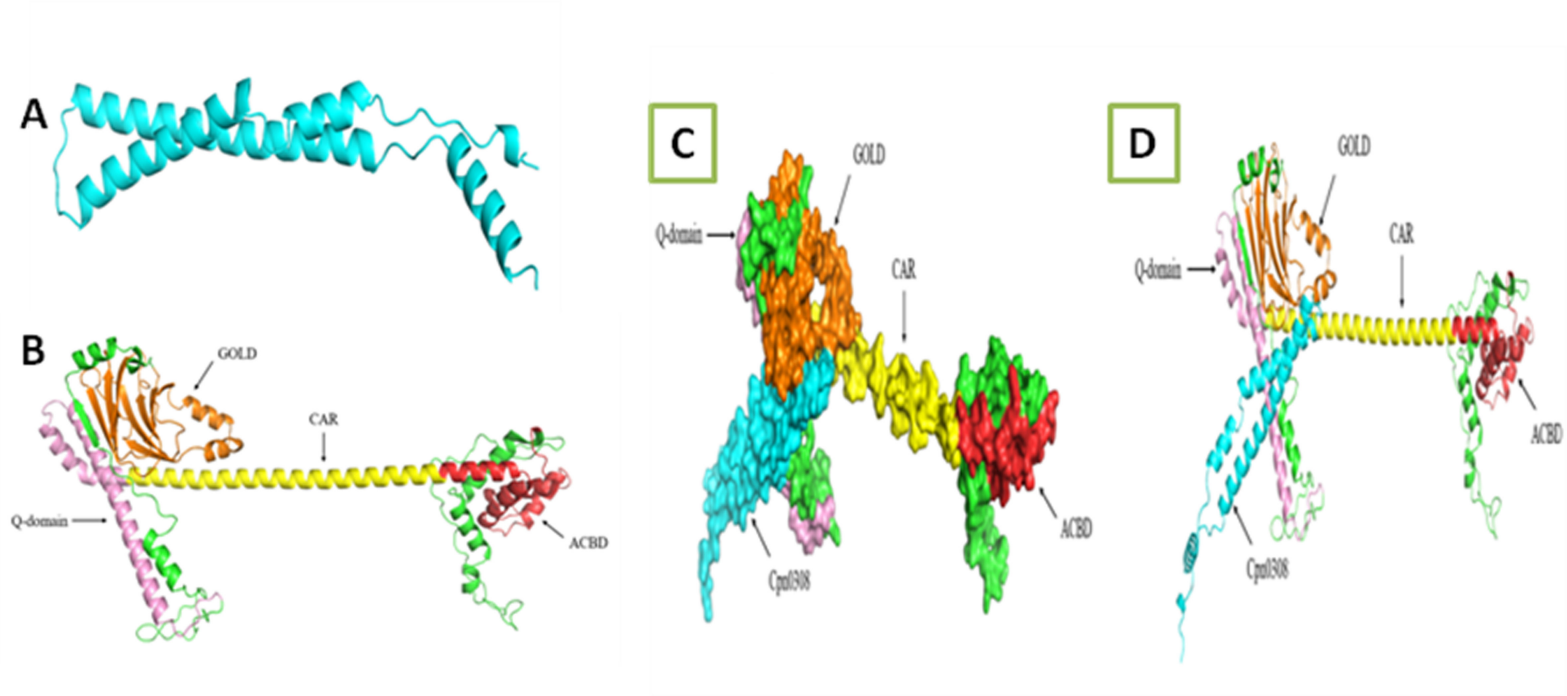
3D structure prediction and docked model of Cpn0308 and ACBD3. The tertial structure models of Cpn0308 (A, cyan) and ACBD3 (B, red for ACB domain, yellow for CAR, pink for Q-domain, and orange for GOLD) were imported into the Vakser Lab online software for constructing the Cpn0308-ACBD3 docking complex model (C and D).

Having demonstrated the chlamydial Cpn0308 interaction with the host cell ACBD3, we next mapped the interaction domain in ACBD3. Ramachandran plot analysis using the PROCHECK program revealed the tertiary structures of both ACBD3 and Cpn0308, with 99.5% of the amino acid residues in the ACBD3 protein model within the allowable range, while 100% in the Cpn0308 protein model. The two models were imported into the Vakser Lab online software for protein docking and to construct the Cpn0308-ACBD3 docking complex model (Fig. 6).

Analysis of the docking model using the PDBePISA online server revealed the amino acid residues involved in the interaction between the two proteins, as well as the bond distances of hydrogen bonds and salt bridges. Three amino acids (R501, E411, and A471) from the GOLD domain of ACBD3 and three amino acids (T47, S54, and Q69) from Cpn0308 are directly involved in the interaction. Thus, the GOLD domain of ACBD3 is responsible for the interaction with Cpn0308.

**Fig 7:**
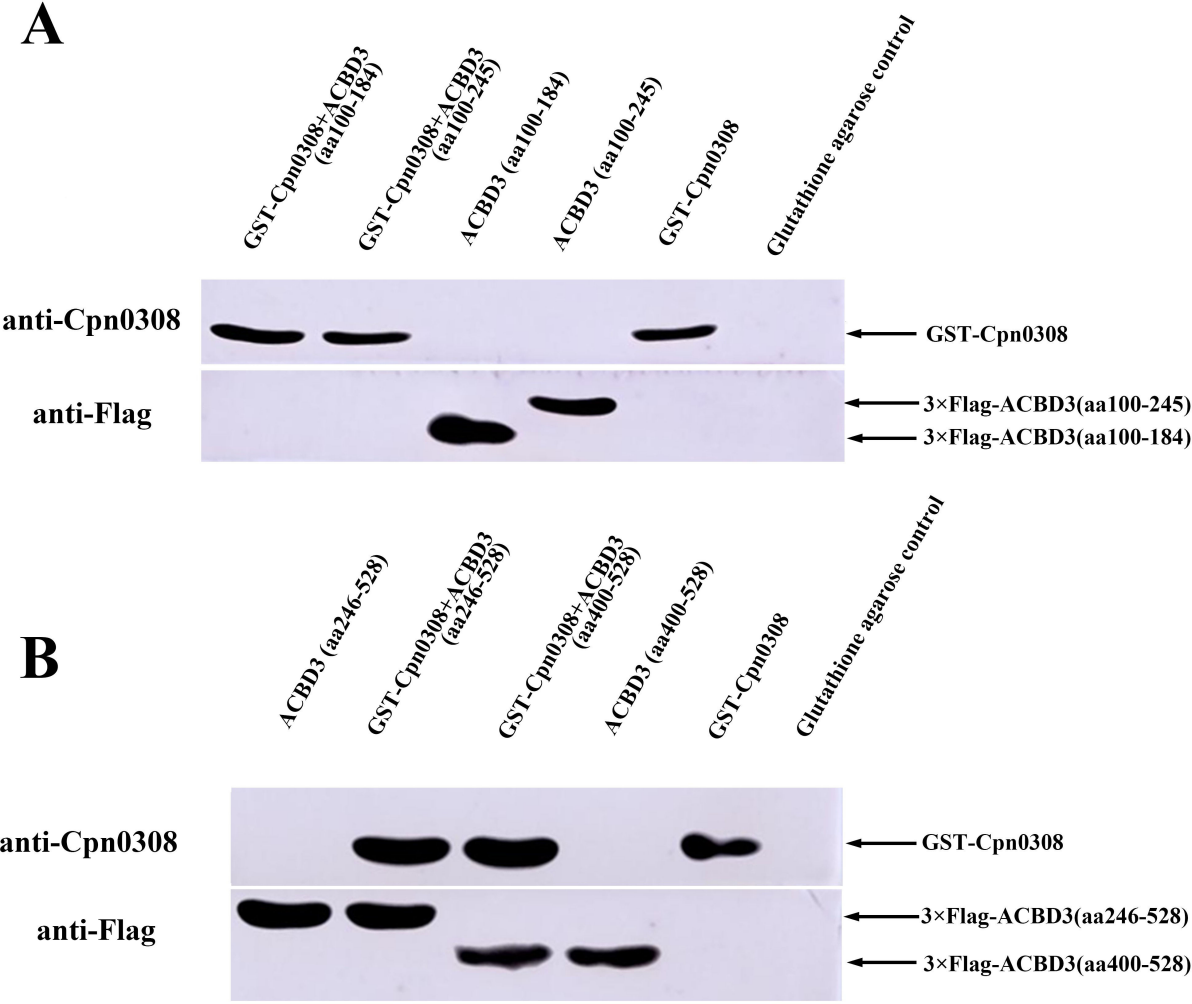
GST-Cpn0308 pull-down of four different ACBD3 fragments. The GST-Cpn0308 was used to pull down each of the four different flag-tagged fragments of ACBD3, which was probed with anti-Flag antibody in western blot. The four Flag-tagged fragments are ACBD3 (100–184) and ACBD3 (100–245) as shown in A and ACBD3 (246–528) and ACBD3 (400–528) in B. Besides the pull-down pellets, the inputs were directly loaded as controls. The complete original experimental image can be found in Fig. S3A and B (Supplementary File).

**Fig 8:**
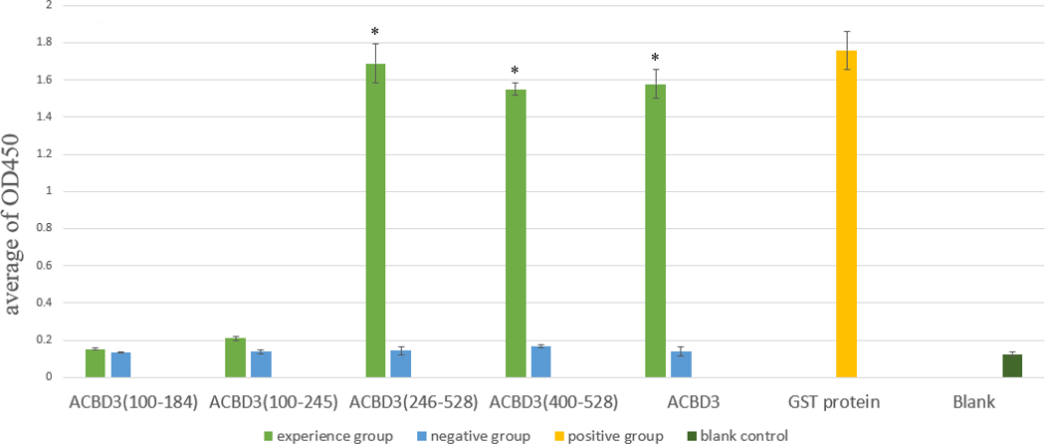
ELISA detection of the GST-Cpn0308 interaction with four different ACBD3 fragments. Flag-tagged ACBD3 fragments immobilized onto a 96-well plate were each used to capture GST-Cpn0308, which was followed by detection with a rabbit anti-Cpn0308 antibody. The binding of GST-Cpn0308 to each Flag-tagged ACBD3 fragment was visualized using an HRP-conjugated goat anti-rabbit secondary antibody plus a soluble substrate. The ELISA reaction plate image was shown (**A**), and the OD values were plotted in **B**. The OD values were significantly higher in wells containing the reactions between GST-Cpn0308 and ACBD3, ACBD3 (246–528), and ACBD3 (400–528), respectively, than those between GST-Cpn0308 and ACBD3 (100–184) and ACBD3 (100–245). **P < 0.01, Student’s *t*-test.

To test whether Cpn0308 interacts with the GOLD domain of ACBD3, we utilized the GST-Cpn0308 fusion protein to pull down different Flag-tagged fragments of ACBD3, including ACBD3 (100–184), ACBD3 (100–245), ACBD3 (246–528), and ACBD3 (400–528). The pellets were detected using an anti-Flag antibody in a western blot analysis (Fig. 7). GST-Cpn0308 pulled down ACBD3 (246–528) and ACBD3 (400–528) but not ACBD3 (100–184) or ACBD3 (100–245), indicating that the GOLD domain (400-528) is sufficient for the interaction with Cpn0308. The GST-Cpn0308 interaction with each of the ACBD3 fragments was also measured using an ELISA (Fig. 8), which validated the results of GST pull-down assay. The above observations provided experimental evidence to validate the computer-predicted results.

## DISCUSSION

*C. pneumoniae* is an obligate intracellular gram-negative bacterium with a unique two-phase development cycle, completing its biosynthesis phase in a cytoplasmic inclusion (22, 23). Some *Chlamydia*-encoded Incs have been shown to mediate the manipulation of host cell machinery and signaling pathways (19, 24, 25). The *C. pneumoniae*-encoded ORF 0308 (Cpn0308) was previously identified as an Inc (15). However, neither its host binding partner nor its function is known. The goal of the current study was to identify the host cell binding partner of Cpn0308. We have provided convincing evidence that Cpn0308 binds to the host cell ACBD3, which is essential for maintaining host cell lipid homeostasis. The current study has at least laid a foundation for further elucidating the mechanisms of Cpn0308 action.

First, we used a Y2H assay to screen Cpn0308 against a HeLa cell cDNA library and identified fatty ACBD3 as a binding partner of Cpn0308. Second, ACBD3 was co-precipitated using an antibody targeting the Cpn0308 fusion protein, which was co-expressed in HeLa cells, indicating that Cpn0308 interacts with ACBD3 within HeLa cells. Third, a GST-Cpn0308 preparation pulled down ACBD3 in a cell-free mixture, which further validating that Cpn0308 binds ACBD3 in an extracellular environment. Fourth, the intracellular interaction between Cpn0308 and ACBD3 was confirmed through microscopy. Fifth, the microscopy was enployed to further visualize the interaction of *C. pneumoniae*-secreted Cpn0308 with ACBD3 endogenously expressed by HeLa cells. Finally, the binding domain in ACBD3 was mapped to the GOLD domain.

ACBD3 is a Golgi multifunctional scaffold protein with 528 amino acid residues, located in the Golgi apparatus (26). ACBD3 can not only maintain the morphology of the Golgi apparatus but also mediate interactions between proteins and participate in the various intracellular signal transduction pathways (27, 28). ACBD3 is structurally divided into the N-terminal ACB region, charged amino acid region (CAR), glutamic acid enrichment region (Q region), and the C-terminal GOLD region (29). The N-terminal ACB domain may interact with acetyl-CoA and its derivatives, playing an important role in the output and sorting of ER proteins; however, its precise function remains unknown (30, 31). The C-terminal GOLD domain is the binding site for interacting proteins and lipids (32). For example, the binding of ACBD3 to giantin in the Golgi apparatus (33) is mapped to residues 373–528 of ACBD3 (29), which is the same region targeted by Cpn0308.

The fact that both giantin and Cpn0308 bind to the same C-terminal GOLD region of ACBD3 suggests that Cpn0308 may compete with giantin for binding to ACBD3. This hypothesis is consistent with our observation that ACBD3, clustered in the Golgi apparatus region, became disseminated in the cytosol after Cpn0308 expression (Fig. 5A and B). Therefore, we speculate that Cpn0308 may compete with giantin for binding to ACBD3 to benefit *C. pneumoniae* intracellular growth. On the other hand, some viral proteins have been shown to interact with ACBD3 to drive the formation of large complexes, such as viral protein/ACBD3/PI4KB (phosphatidylinositol-4-kinase B), following viral infection (32). Regardless of whether Cpn0308 binding to ACBD3 to dissociate it from the Golgi apparatus or drive the formation of even larger complexes, we hypothesize that Cpn0308 binding to the GOLD domain of ACBD3 may allow *C. pneumoniae* to access more lipids from the Golgi apparatus. It will be interesting to test whether brefeldin A treatment, which is known to block Golgi lipid trafficking to *Chlamydia* (34, 35), can inhibit the function of Cpn0308. This and other mechanisms of interest will be evaluated once funding is secured.

## Data Availability

The data sets generated during this study are available from the corresponding author upon reasonable request.

## SUPPLEMENTAL MATERIAL

Supplemental figures jb.00275-24-s0001.pdf Fig. S1 to S3.

ASM does not own the copyrights to Supplemental Material that may be linked to, or accessed through, an article. The authors have granted ASM a non-exclusive, world-wide license to publish the Supplemental Material files. Please contact the corresponding author directly for reuse.
