# Supplementary material for: The Chlamydia pneumoniae inclusion membrane protein Cpn0308 interacts with host protein ACBD3
Source: J Bacteriol. 2024 Dec 26;207(1):e00275-24. doi: 10.1128/jb.00275-24 (PMC11784219; doi:10.1128/jb.00275-24)
Supplement: Supplemental figures — Fig. S1 to S3. [file jb.00275-24-s0001.pdf]

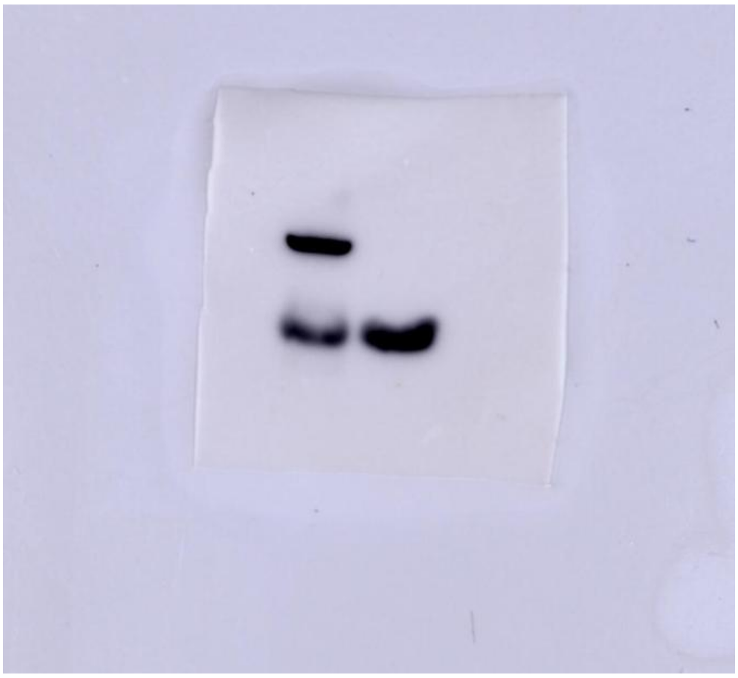

Fig. S1 The complete original experimental image of the immunoprecipitation experiment of the Cpn0308-ACBD3 interaction complex

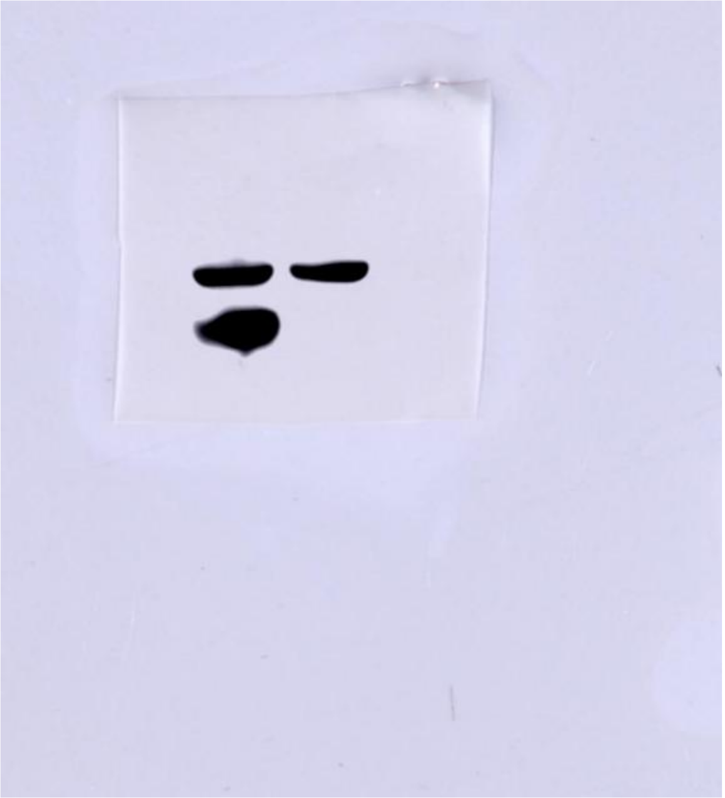

Fig. S2 The complete original experimental image of GST-Cpn0308 pull-down of ACBD3

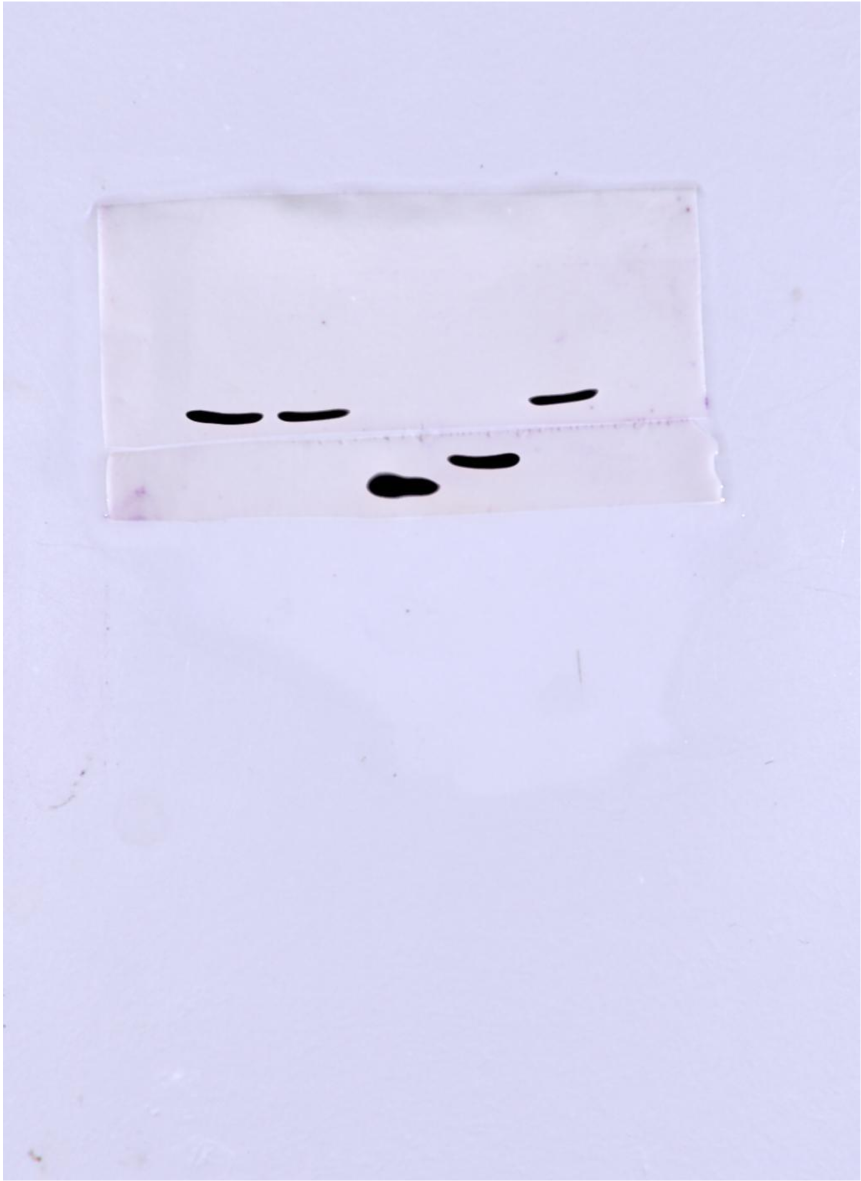

Fig. S3-A The complete original experimental image of GST-Cpn0308 pull-down of four different ACBD3 fragments.

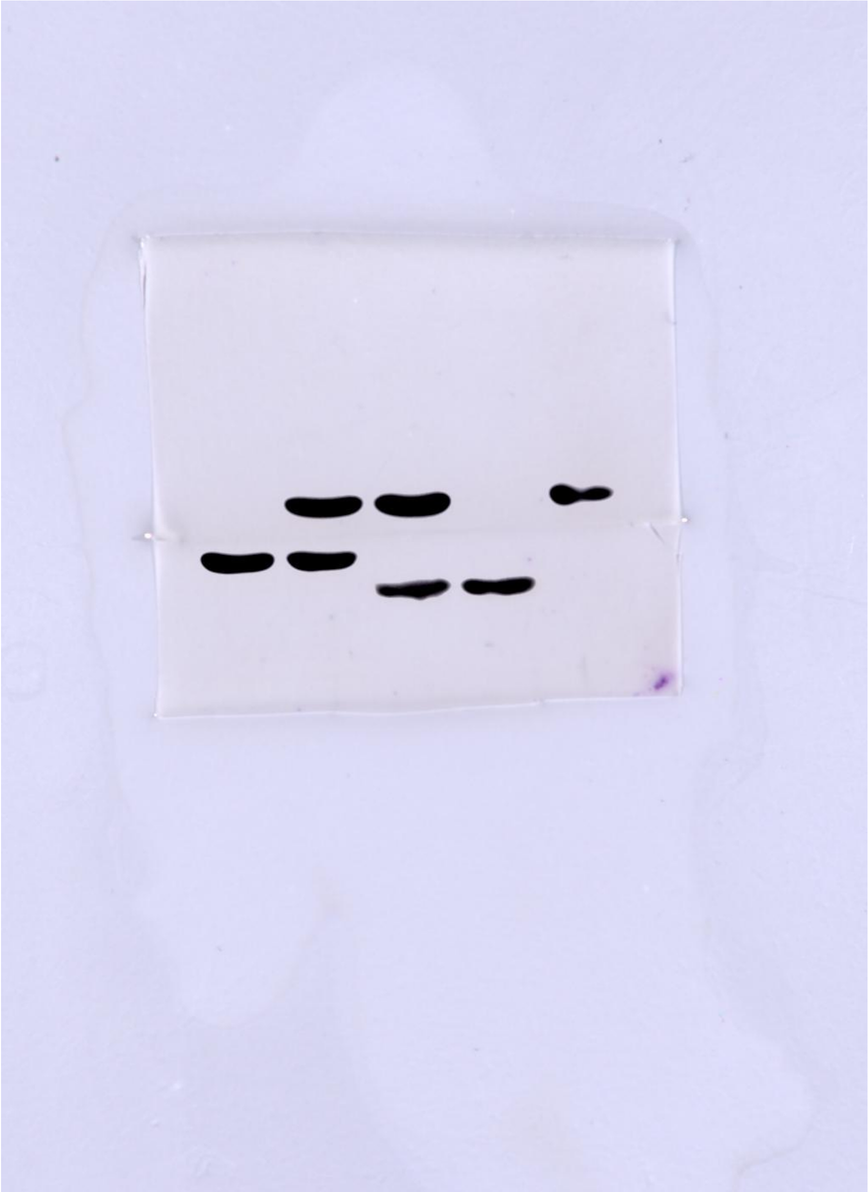

Fig. S3-B The complete original experimental image of GST-Cpn0308 pull-down of four different ACBD3 fragments.
